# Supplementary material for: AncesTree: An interactive immunoglobulin lineage tree visualizer
Source: PLoS Comput Biol. 2020 Jul 10;16(7):e1007731. doi: 10.1371/journal.pcbi.1007731 (PMC7375605; doi:10.1371/journal.pcbi.1007731)
Supplement: Attachment — Submitted filename: AncesTree_review_visual_bug.pdf [file pcbi.1007731.s001.pdf]

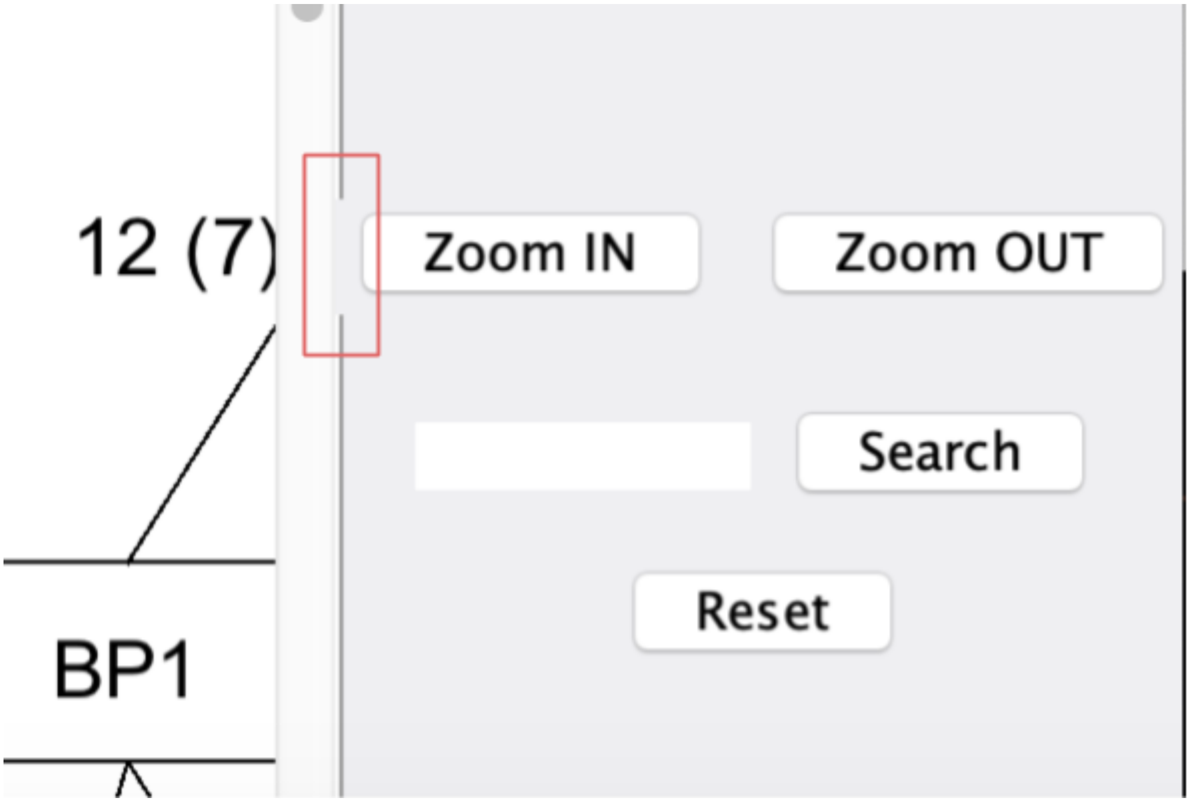
12 (7)
BP1
Zoom IN
Zoom OUT
Search
Reset

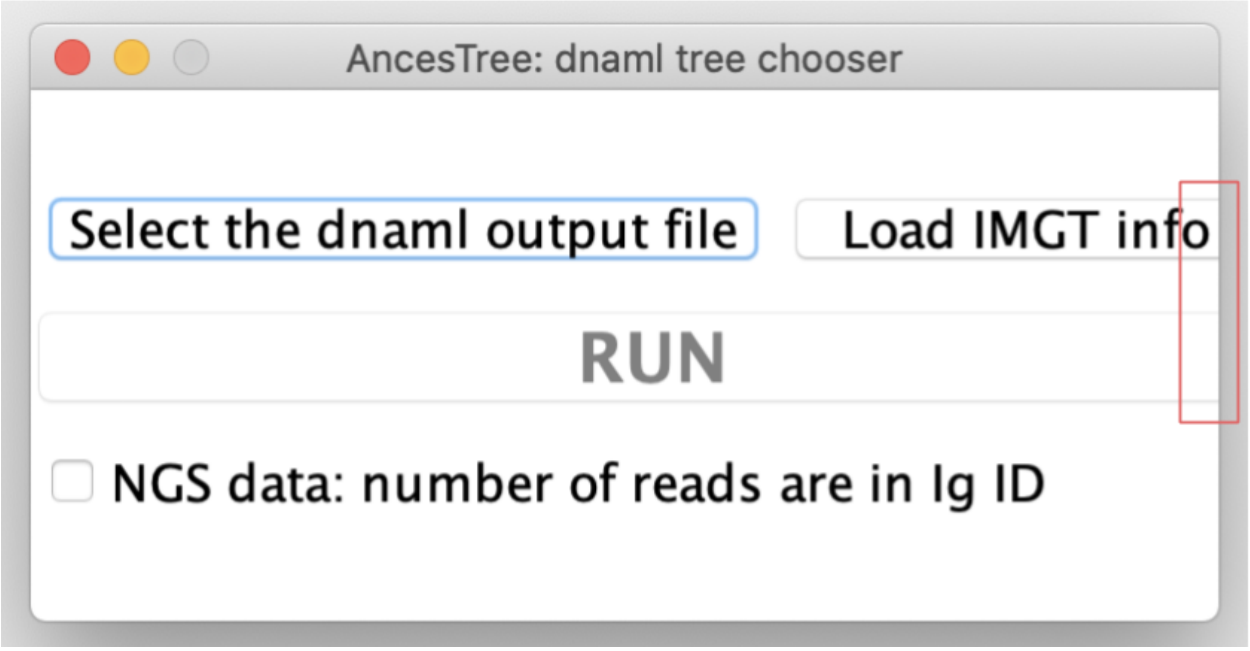
AncesTree: dnaml tree chooser
Select the dnaml output file
Load IMGT info
RUN
NGS data: number of reads are in Ig ID

Amino acid sequence:	QVQLVQSGAEIKKPGSSVKVSCRASGTTFRSFAISWVRQA<br>PGQGLEYMGGVIALFGTANYAQKFQGRVTITADESTNTAY<br>MELSSLRSEDTAVYYCAKGSGYHVRDYFDYWGQGTLVTVS<br>S
EC 50 (ng/ml):	*Enter the EC 50 here*
Comment 1	Show
Comment 2	
