# Supplementary material for: AncesTree: An interactive immunoglobulin lineage tree visualizer
Source: PLoS Comput Biol. 2020 Jul 10;16(7):e1007731. doi: 10.1371/journal.pcbi.1007731 (PMC7375605; doi:10.1371/journal.pcbi.1007731)
Supplement: Attachment — Submitted filename: Rebuttal_letter_Foglierini_et_al.docx [file pcbi.1007731.s002.docx]

18^th^ May 2020,

Mihaela Pertea, PhD

Software Editor, PLOS Computational Biology

Dear Dr. Pertea,

Thank you for the reviews of our manuscript entitled “'AncesTree: an interactive immunoglobulin lineage tree visualizer”, which has been revised for PLOS Computational Biology, as per your detailed guidance.

We thank the Reviewers for their very helpful feedback. As outlined below, we have made extensive efforts to address all of the Reviewer’s concerns and suggestions. The revised version of the manuscript contains a description of the new features incorporated in the software, such as compatibility to other AIRR data processing packages that strengthen the utility of our software. All the figures have been revised accordingly to incorporate the new results and address the reviewer’s suggestions and comments. As a result, we believe that our revised manuscript is significantly improved.

We are looking forward to hearing from you.

Sincerely,­­

**Mathilde Foglierini**

Institute for Research in Biomedicine

**Responses to Reviewers**

We thank the Reviewers for their extremely helpful feedback on our manuscript and several suggestions for improvements. We have addressed all major and minor points, and edited the manuscript following the Reviewers suggestions. As a result, we believe that our revised manuscript and software are significantly improved.

Please note that the Reviewer comments are in black, quoted exactly as provided, and our responses are in blue.

**Reviewer #1:**

***Comments to the Authors:
Please note here if the review is uploaded as an attachment.*** *Reviewer #1: The authors present a graphical user interface for visualizing B cell clonal lineage trees. The program takes results from Phylip, a computational phylogenetics package for inferring evolutionary trees, as an input and allows the user to visually explore the constructed trees by providing features such as annotation of somatic hypermutations or IC50 on the tree edges and annotation of tree nodes (i.e. Ig sequences) with IMGT and BASELINe tools.
The interface provides a good user experience in terms of available features but lacks broader support of alternative B-cell phylogenetic inference tools.
The program is being distributed as a .jar-package which makes it easy to install and run on the provided tutorial data.*

*Reviewer #1 was supportive, as reflected in his/her summary statements: “The interface provides a good user experience in terms of available features”.*

*The reviewer also had several comments, questions, and suggestions to improve the manuscript.*

*Major issues:
- The authors built their interface upon Phylip, which is indeed a widely used tool for constructing phylogenetic trees. However, adding support of other AIRR data processing packages (such as Immcantation, including Change-O and IgPhyML that also uses Phylip algorithm) would greatly increase the number of potential users that can profit from this great UI. Indeed, the AncesTree should be able to run on any tree independent of how the tree was constructed. Thus, the importance given to phylip, especially in the abstract, is not understandable.*

*- An issue that can hinder the use of the program is the need for a user to decompose the repertoire into clonal lineages by themselves prior to Ancestree analysis, which may be done by Change-O or Clonify. Why is the lineage decomposition not provided out of the box by Ancestree?*

**Response: Addressed.** Reviewer #1, as well as Reviewer #2, rightly points out that Ancestree was not adding support to AIRR data processing packages. We have modified the software and Ancestree is now able to support input coming from the Immcantation workflow: Change-O and IgPhyML files can now be used as AncesTree input files. The Change-O file has to be in AIRR format thus supporting a standard format for sharing AIRRseq data. Output from IgPhyML produces a tab file with the clone identifiers and their related trees in newick format, together with a fasta file with the reconstructed intermediate sequences. The fasta file is needed for AncesTree (--asr option will produce this file). The information about FR/CDR regions are parsed directly from the Change-O file. Therefore, once the user run an Immcantation workflow with the “classical steps” of AIRR seq analysis such as raw reads processing, VDJ annotation, VDJ reference alignment, clonal clustering, germline inferring and tree reconstruction, the use of AncesTree is straightforward. This option is now specified in the text and shown in the new Figure 1.

Regarding the clonal lineage assignment step, we added several missing references, including Clonify, in the introduction of the manuscript (lanes 47-54).

*- The link to the wiki* [*https://bitbucket.org/mathildefog/ancestree/wiki/*](https://bitbucket.org/mathildefog/ancestree/wiki/) *is closed: a user from the outside would not have access to it.*

**Response: Addressed.** We apologize for the closed wiki link. It is now open and available without restriction on the following link: <https://github.com/MathildeFogPerez/ancestree/wiki>. In addition, we moved all the source code, executable, wiki and examples to <https://github.com/MathildeFogPerez/ancestree>.

*- The graphical interface has a lot of minor visual bugs. Each of the bugs is not critical on its own, but in total, they spoil the impression that the appearance of the interface leaves (see attachment).*

**Response:** We thank Reviewer #1 for notifying us about the visual bugs. Unfortunately, AncesTree was intensively tested on Windows and Linux OS, but to a lower extend on MAC OS. This issue is specific to MAC OS and we couldn’t identify the reason behind it.

*- The authors did not include a quantification of how large datasets may be to be feasibly analysed with AncesTree?*

**Response:** **Addressed**. We agree with Reviewer #1 that we should have specified the datasets size that can be used in AncesTree. We tested AncesTree with a clonal family of 1248 sequences (not all unique) processed through Dnaml. Moreover, using change-O/IgPhyML workflow, we tested several clonal families with a minimum of 300 sequences (all unique using the collapse option). We did not identify a limiting dataset size.

*- Figure 3 is not a data figure (or unrelated to the overall purpose of the paper) and should therefore be merged with Figure 4 or moved to Supplementary.*
**Response: Addressed.** We agree with Reviewer #1, and this figure was merged with Figure 4, thus becoming new Figure 3. *Minor issues:
- The trees in the tutorial video and the example data differ.*

**Response: Addressed.** We agree and we have modified the video. In addition, we also show how to create the tree with different inputs. The video is now available on YouTube: <https://www.youtube.com/watch?v=i2ooM1eLnBk&feature=youtu.be>.

*- The manuscript text requires language editing (many typos/grammar mistakes)*

**Response:** **Addressed**. We thank Reviewer#1 for notifying us about the typographic mistakes present in our manuscript. We have corrected the text throughout the manuscript.

*- Add reference to change-o*
**Response: Addressed.** Reference to change-o is now present in the manuscript.

**Reviewer #2:**

*Reviewer #2: The authors of "AncesTree: an interactive immunoglobulin lineage tree visualizer" describe a software tool aimed at facilitating the analysis of B-cell receptor (BCR) evolution and exploration of BCR lineage trees. I find this tool to be of high utility for the community, especially immunologists with little or no computational skills. While I find this tool quite versatile, it seems there is an issue with supported input formats.*

Reviewer #2 was also supportive, as reflected in his/her summary statements: “I find this tool to be of high utility for the community, especially immunologists with little or no computational skills.”

The reviewer had also several questions and suggestions for the manuscript.

*1) There is a standard coming from AIRR community [*[*https://docs.airr-community.org/en/latest/*](https://docs.airr-community.org/en/latest/)*] that provides a comprehensive description of T- and B-cell receptor repertoire sequencing data. In order to be useful for a wider community I suggest incorporating support for AIRR format in the software.*

**Response: Addressed.** Please see answer to Reviewer #1 comment.

*2) In line with previous point, tools like MIXCR and IgBlast are widely used to analyze large amounts of B-cell receptor sequencing data, while IMGT web server is limited to a certain number of input reads and is unfeasible for large sequencing datasets. Authors should consider implementing a mean to import results produced by MIXCR and IgBlast.*

**Response: Addressed.** We agree with Reviewer #2 that AncesTree could not depend only on IMGT web server for the Ig sequences annotation. Therefore, while adding the Change-O files as input to AncesTree, we allow the user to use not only IMGT/high-VQUEST webserver but also IgBlast for the Ig sequences annotation. We did not include MiXCR as import option for AncesTree for two reasons. First, MiXCR is not supported as a Change-O input. Second, because MiXCR alone does not provide phylogenic inference, so it cannot be visualized.

Our goal with AncesTree is to provide the user with two kinds of analysis: one based on RepSeq data with a large variety of trees to analyze, this is now possible using the Immantation framework. The second type of analysis is based on a clonal family of interest, this is the case of immunologist identifying and Sanger sequencing some antibodies based on their functions. In the aforementioned case, the scientist could do the analysis without any computational skills, which is not the case if you analyze RepSeq data.

*3) There is no comparison with Immcantation suite [*[*https://immcantation.readthedocs.io/en/stable/*](https://immcantation.readthedocs.io/en/stable/)*], nor it is mentioned in discussion. This suite is widely used for B-cell lineage analysis so authors should comment on AncesTree features that are not present in this software.*

**Response: Addressed.** Please see answer to Reviewer #1 comment.
